# DNA polymerase iota (Pol ι) promotes invasion and metastasis of esophageal squamous cell carcinoma

**DOI:** 10.18632/oncotarget.8580

**Published:** 2016-04-04

**Authors:** Shitao Zou, Zeng-Fu Shang, Biao Liu, Shuyu Zhang, Jinchang Wu, Min Huang, Wei-Qun Ding, Jundong Zhou

**Affiliations:** ^1^ Suzhou Cancer Center Core Laboratory, Nanjing Medical University Affiliated Suzhou Hospital, Suzhou, Jiangsu, 215001, P.R. China; ^2^ School of Radiation Medicine and Protection, Medical College of Soochow University, Collaborative Innovation Center of Radiation Medicine of Jiangsu Higher Education Institutions, Suzhou, Jiangsu, 215123, P.R. China; ^3^ Department of Pathology, University of Oklahoma Health Science Center, Oklahoma City, OK 73104, USA

**Keywords:** esophageal squamous cell carcinoma, DNA polymerase iota, tumor metastasis, JNK-AP-1 cascade, nude mouse

## Abstract

DNA polymerase iota (Pol ι) is an error-prone DNA polymerase involved in translesion DNA synthesis (TLS) that contributes to the accumulation of DNA mutations. We recently showed that Pol ι is overexpressed in human esophageal squamous cell cancer (ESCC) tissues which promotes ESCC' progression. The present study was aimed at investigating the molecular mechanisms by which Pol ι enhances the invasiveness and metastasis of ESCC cells. We found that the expression of Pol ι is significantly higher in ESCCs with lymph node metastasis compared to those without lymph node metastasis. Kaplan-Meier analysis revealed an inverse correlation between Pol ι expression and patient prognosis. The expression levels of matrix metalloproteinase-2 (MMP-2) and matrix metalloproteinase-9 (MMP-9), two essential regulators of cells' invasiveness, were positively associated with Pol ι expression in ESCC tissues. Ectopic expression of Pol ι enhanced the motility and invasiveness of ESCC cells as evaluated by wound-healing and transwell assays, respectively. A xenograft nude mouse model showed that Pol ι promotes the colonization of ESCC cells in the liver, lung and kidney. Signaling pathway analysis identified the JNK-AP-1 cascade as a mediator of the Pol ι-induced increase in the expression of MMP-2/9 and enhancement of ESCC progression. These data demonstrate the underlying mechanism by which Pol ι promotes ESCC progression, suggesting that Pol ι is a potential novel prognostic biomarker and therapeutic target for ESCC.

## INTRODUCTION

Esophageal cancer is the sixth most common cause of cancer-related death worldwide [1]. Esophageal squamous cell carcinoma (ESCC) is the major histological type of esophageal cancer (>90%) in China and accounts for 60-70% of esophageal cancer cases worldwide [2, 3]. Despite the improvement of diagnosis and treatment throughout the past decades, the management of ESCC remains a challenge due to its rapid progression and metastasis [4]. Cancer metastasis is a complex multi-step process involving in uncontrolled proliferation, migration, invasion, adhesion and angiogenesis [5]. Among them, invasion is recognized as one of the fundamental characters of malignant tumor cells, which enables the cells to overcome the obstacles of cell-extracellular matrix (ECM) boundary and invade into lymph and blood vessels [6, 7]. The lymph node invasion is considered one of the most important indicators for poor prognosis of ESCC patients [8, 9]. Although a number of molecular alterations have been identified, the mechanism by which cancer cells invade to lymph nodes has not been fully elucidated. Identification of specific molecular indicators relating to lymph node invasion will provide novel prognostic and therapeutic strategies against metastatic ESCC.

The genome is continuously exposed to various damaging factors coming from endogenous cell metabolism or exogenous insults, such as UV light and other genotoxic agents. These stresses may cause DNA damage and subsequently increase the genomic instability (GIN) of cells. GIN is one of the most important features and driving forces for cancer development [10]. To protect the integrity of genome, cells have evolved repair mechanisms to correct specific types of DNA damage [11]; however, some lesions cannot be completely eliminated and will remain in DNA. The replication of unrepaired DNA will result in the stalling of high fidelity polymerases at the replication fork, named replication stress, ultimately leading to replication fork collapse and an increase in the frequency of chromosomal translocations and aberrations. Fortunately, cells employ the translesion DNA synthesis (TLS) system that recruits a group of low fidelity DNA polymerases to bypass the altered DNA template [12, 13]. Based upon the structural homology, the TLS polymerase is classified into Y-family (pol ι, pol κ, pol η and Rev1) and B-family (pol ζ), which commonly exhibit error-prone nature during DNA synthesis [13]. Among them, Pol ι has the lowest fidelity to bypass many types of DNA lesions during the TLS process and can also generate mutations when replicating undamaged DNA.

Consistent with its important role in the TLS pathway and its error-prone character, Pol ι was considered a double-edged sword guarding the genome and regulating cancer progression [12, 14]. Several previous studies revealed that the deficiency of Pol ι increases susceptibility to urethane-induced lung adenomas [15–17]. Ohkumo and colleagues showed that loss of Pol ι slightly accelerates the process of UV-induced mouse skin epithelial tumors under a *Polh−/−* background and that *Polι*−/− mice are more likely to form mesenchymal tumors, such as sarcomas, after UV exposure [18]. Pan et al demonstrated that the expression of Pol ι is downregulated in human stomach, lung and colorectal cancers [19]. On a flip side, however, overexpression of Pol ι may contribute to the accumulation of genomic mutations, subsequently increasing the frequency of a tumor acquiring phenotype. For instance Malkas' group identified an elevated expression of Pol ι in human breast cancer cells, which was correlated with decreased DNA replication fidelity [20]. We have recently demonstrated that the expression of Pol ι is upregulated in ESCC tissues [21, 22] and that the dysregulation of Pol ι harasses cell cycle progression through enhancing cyclin D1 expression [22]. However, the relationship between Pol ι expression and tumor invasion and metastasis of ESCC remains unclear. Here, we compared the expression pattern of Pol ι in clinical samples and uncovered the promoting role of Pol ι in ESCC lymph node metastasis. Furthermore, we found that overexpression of Pol ι increases the transcription of the matrix metalloproteinase (MMPs) genes by activating the JNK-AP-1 pathway. These data suggest that Pol ι is a novel biomarker for tumor metastasis in ESCC.

## RESULTS

### Pol ι expression correlates with lymph node metastasis and poor prognosis in human ESCC

**Figure 1:**
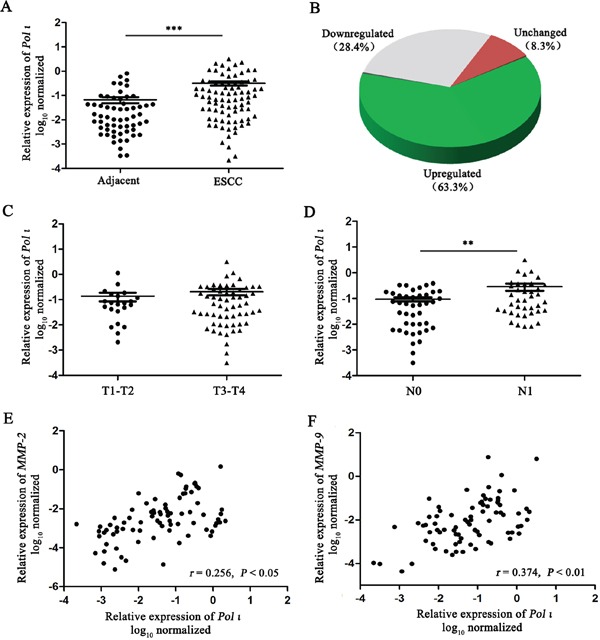
Pol ι expression is frequently increased in human ESCC **A-B.** Pol ι expression was examined by qRT-PCR in 82 ESCC tissue samples and 60 matched adjacent normal esophageal tissue samples. Pol ι expression was significantly increased in tumor tissues compared with that in adjacent normal esophageal tissues (***p<0.001, Student's t test). **C-D.** The correlation between Pol ι expression and T stages (C, *p*>0.05) or lymph node metastasis (D **p<0.01, Student's t test). **E-F.** MMP-2 and MMP-9 expression were examined by qRT-PCR in 82 ESCC tissue samples, and Pol ι expression was positively correlated with the MMP-2 (*p*<0.05, Pearson's correlation) or MMP-9 expression (*p*<0.01, Pearson's correlation).

Our previous studies demonstrated that Pol ι is highly expressed in ESCC tissues compared to adjacent normal tissues [22]. To further confirm this observation, the expression of *Pol ι* mRNA was examined with qRT-PCR in an expanded cohort of 82 ESCC tissue samples and 60 matched adjacent normal esophageal tissue samples. As shown in Figure 1A and 1B, Pol ι expression was significantly increased in ESCC tissues (63.3%) compared with adjacent normal esophageal tissues (*p*<0.001). Lymph node metastasis has been characterized as the single most important prognostic factor for esophageal cancer [23]. Our results revealed that the expression of Pol ι is significantly higher in ESCCs with lymph node metastasis than those without lymph node metastasis (*p*<0.01, Figure 1D), although the expression level is not correlated with T stages (*p*>0.05, Figure 1C).

The increased expression of MMPs is necessary for tumor invasion and metastasis due to their role in degrading the cell-extracellular matrix. Our previous investigation showed that there seems to be no relationship between *Pol ι* and *MMP* expression in tumor tissues, based on a relatively small cohort [22]. However, when we compared the expression levels of *Pol ι* and *MMP-2/9* in ESCC using a larger clinical sample size (n=82), the results showed that the expression levels of *Pol* ι and *MMP-2/9* are positively correlated in ESCC (*p*<0.05, Figure 1E and 1F). These results suggest that Pol ι may promote tumor progression through enhancing ESCC invasion and metastasis.

**Figure 2:**
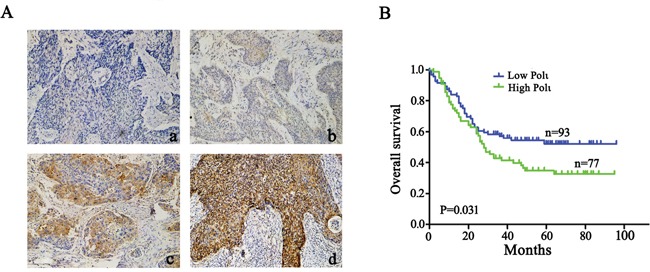
Pol ι expression correlates with poor prognosis in human ESCC **A.** immunohistochemical staining of Pol ι in 170 tumor tissue samples (100×), a: Negative expression; b, Low expression; c, Moderate expression; d, High expression. **B.** survival analysis based on the expression level of Pol ι. The groups were ranked according to the Pol ι staining intensity. The percentages of overall survival in low Pol ι expression (A, a-b) was significantly higher than that of patients with high Pol ι expression (A, c-d) (*p*=0.031).

We next analyzed Pol ι protein expression in 170 ESCC patients by immunohistochemical assay. Immunohistochemical staining combined with Kaplan-Meier survival analysis revealed that the patients with higher Pol ι expression in ESCC tissues has poorer clinical outcomes (*p*=0.031; Figure 2A and 2B), indicating that Pol ι protein expression is positively associated with tumor progression of ESCC

### Pol ι promotes ESCC cell migration and invasion *in vitro*

**Figure 3:**
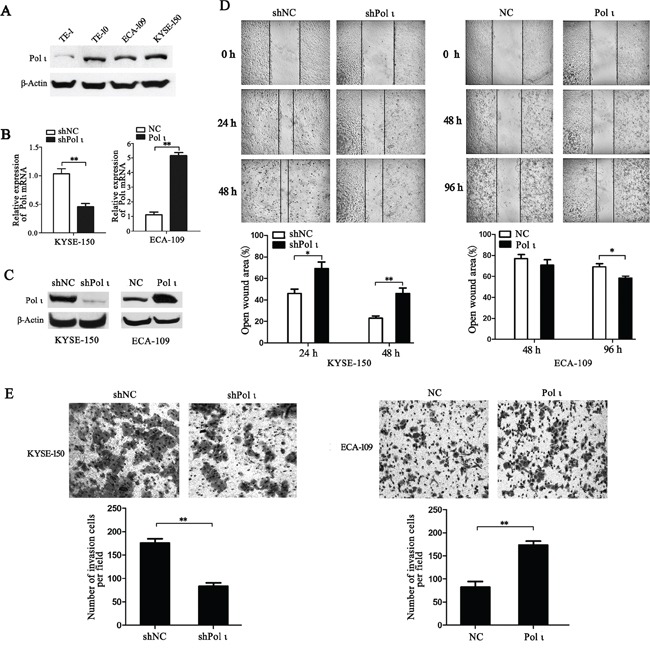
Pol ι promotes ESCC cell migration and invasion *in vitro* **A.** western blot was performed to detect the expression of Pol ι in four ESCC cell lines. **B-C.** Pol ι overexpression and knockdown cell lines were constructed using LV-EGFP vectors. qRT-PCR (B) and western blot (C) were used to analyze Pol ι expression in these cell lines.β-actin levels served as loading control. **D.** the wound-healing assay was performed to examine the cell migration. Cells were seeded in 6-well plates that reached confluence in 24 h. Wound healing was measured at 0, 24, 48 and 96h after scratch. The open wound area was normalized to the area at the initial time (0 h). Pol ι knockdown repressed the cell migration of KYSE-150 cells, whereas overexpression of Pol ι enhanced the cell migration of ECA-109 cells (*p < 0.05, **p<0.01, Student's t test). **E.** The matrigel transwell assay was performed to determine cell invasion potential. 1×10^5^ cells were plated in transwell inserts and incubated for 24 or 48 h. Cells migrated to the bottom were stained with crystal violet and calculated manually. Pol ι knockdown repressed the cell invasion of KYSE-150 cells, and overexpression of Pol ι enhanced the cell invasion of ECA-109 cells (**p<0.01, Student's t test).

To understand how Pol ι is associated with ESCC invasion, ectopic expression (ECA-109-Polι compared with ECA-109-NC) and specific knockdown of Pol ι (KYSE-150-shPolι compared with KYSE-150-shNC,) in ESCC cell lines were achieved. We chose ECA-109 for overexpression and KYSE-150 for knockdown study due to the fact that Pol ι is significantly highly expressed in KYSE-150 cells compared to ECA-109 cells. Western blotting and quantitative real-time PCR confirmed the expression levels of Pol ι in these cell lines (Figure 3A-3C). As increased motility is essential for tumor metastasis, Pol ι-overexpressing and -knocking down cells were first subjected to *in vitro* wound-healing assays. Confluent cell cultures were scraped to give rise to a wound, and cell motility was determined at different time points (24h and 48h for KYSE-150 cells; 48h and 96h for ECA-109). As shown in Figure 3D, overexpression of Pol ι in ECA-109 cells dramatically narrowed the wound area as compared to the control cells, whereas Pol ι depletion slowed down the migration of KYSE-150 cells. Since invasion is also an important step for cancer cell metastasis, we performed the Boyden chamber transwell assay to explore the effect of altered Pol ι expression on the invasiveness of ESCC cells. As shown in Figure 3E, the expression levels of Pol ι were positively correlated with the ability of ESCC cells to invade through the Matrigel coated membrane. Collectively, these data indicated that Pol ι promotes migration and invasion of ESCC cells.

### Knockdown of Pol ι decreases the metastatic potential of ESCC cells *in vivo*

**Figure 4:**
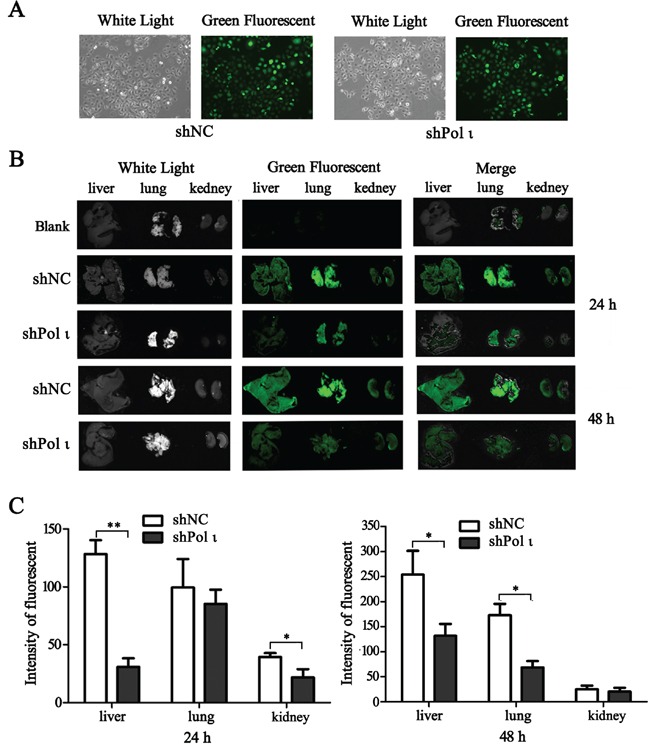
Knockdown of Pol ι expression decreases the colonization of KYSE-150 cells *in vivo* **A.** the particles containing *Pol ι* shRNA-EGFP vectors were used to infect KYSE-150 cells, and the stable cell lines were selected in medium containing 1μg/ml Puromycin for 7 days. **B.** cells were injected into nude mice via the tail vein. After 24 or 4 h, the livers, kidneys and lungs of the mice were removed and KYSE-150 cell colonization in the organs was visualized using an *in vivo* imaging system. All the photos were taken under the same condition. **C.** relative green fluorescence levels in the indicated group of tissues were calculated using the ImageJ image analysis software (*p < 0.05, **p<0.01, Student's t test).

**Figure 5:**
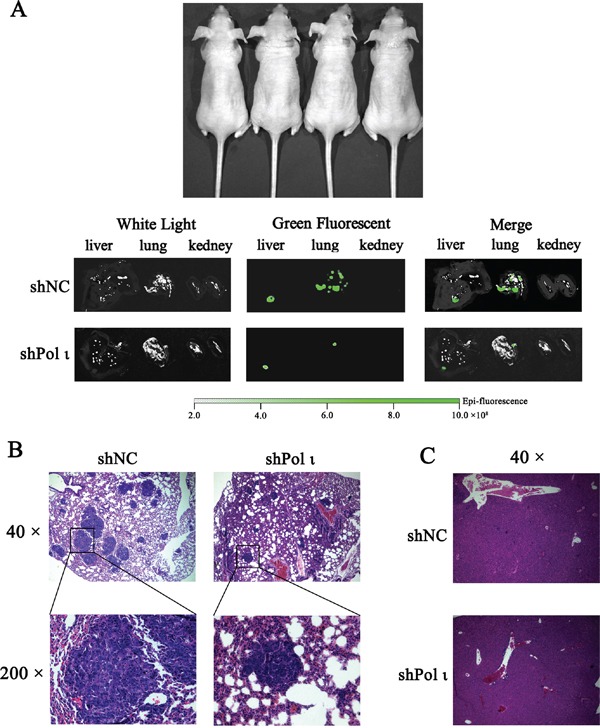
Knockdown of Pol ι expression decreases the metastatic potential of KYSE-150 cells *in vivo* Cells were injected into nude mice via the tail vein. **A.** after 30 days, the livers, kidneys and lungs of nude mice were removed and the metastatic status of transplanted KYSE-150 cells into those organs was evaluated by an *in vivo* imaging system. **B-C.** the lungs (B) and livers (C) were fixated in 10% phosphate-buffered formaldehyde and embedded in paraffin. Tumors were confirmed in H&E-stained sections.

We further tested the effect of Pol ι depletion on the metastatic potential of ESCC cells *in vivo*. KYSE-150 cells, infected with the lentivirus containing either the control shRNA or specific *Pol ι* shRNA, were inoculated into nude mice via the tail vein. Both cell lines expressed LV-EGFP (Figure 4A), thus allowing us to monitor the cells *in vivo*. KYSE-150 cells colonization in different mouse organs were detected using an *in vivo* imaging system at 24h, 48h and 30 days after inoculation. As shown in Figure 4B, KYSE-150-shPolι cells exhibited a weaker signal of green fluorescence in the livers, lungs and kidneys of mice compared with the control KYSE-150 groups (Figures 4B and 4C), indicating that KYSE-150-shPolι cells are less colonized in these organs. The difference of green fluorescence levels, especially in the livers and lungs, was even more pronounced between the two groups of mice after 30 days of inoculation (Figure 5A). The metastatic status of transplanted KYSE-150 cells into lung and liver was further evaluated by H&E staining. As shown in Figure 5B, knocking down Pol ι expression dramatically decreased the number and size of tumors in lungs, and left no tumors in livers (Figure 5C). Taken together, these results indicated that downregulation of Pol ι expression significantly inhibits the metastatic potential of ESCC cells *in vivo*.

### Pol ι upregulates the expression of MMP-2 and MMP-9 through activating the JNK-AP-1 pathway in ESCC cells

**Figure 6:**
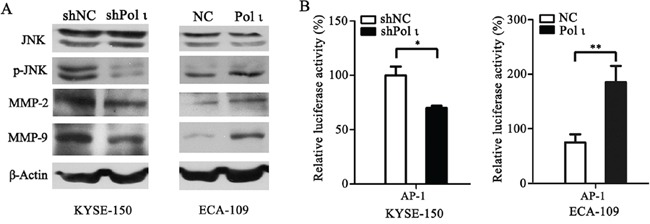
Pol ι enhances MMP-2 and MMP-9 expression through activating the JNK-AP-1 pathway **A.** western blot analysis of the expression levels of JNK, p-JNK, MMP-2 and MMP-9 in Pol ι overexpression and knockdown cell lines.β-actin levels served as loading control. **B.** cells were seeded in 24-well plates and transfected with pAP1-Luc vector for 36h. The luciferase activity was analyzed by a dual-luciferase reporter system. The luciferase activity was normalized by co-transfection with 50 ng pRL-SV40 vector and analysis of Ranilla activity (*p < 0.05, **p<0.01, Student's t test).

**Figure 7:**
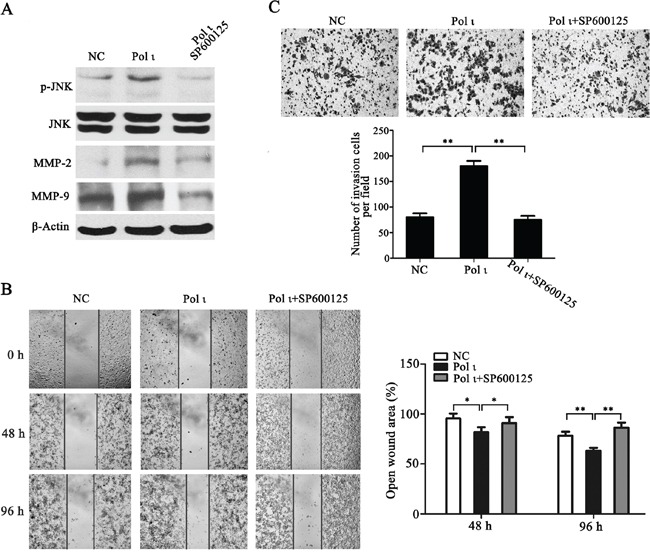
Pol ι promotes ESCC cell migration and invasion through the JNK-AP1 pathway Pol ι overexpression cells were pretreated with SP600125 for 24h. **A.** western blot analysis of the expression levels of JNK, p-JNK, MMP-2 and MMP-9. **B.** wound-healing assay at 0, 48 and 96h after scratch. The open wound area was normalized to the area at the initial time (0 h, *p < 0.05, **p<0.01, Student's t test). **C.** cells were plated in matrigel transwell inserts and incubated for 48h. Cells migrated to the bottom were stained with crystal violet and calculated manually (**p<0.01, Student's t test).

MMPs induce extracellular matrix remodeling and promote cancer cell invasion and metastasis [24]. To understand the potential mechanisms of Pol ι-mediated tumor invasion and metastasis, we examined the effect of altered Pol ι levels on MMP-2 and MMP-9 expression in our model systems. Overexpression of Pol ι increased MMP-2 and MMP-9 expression in ECA109 cells as evidenced by western blot analysis. In contrast, specific knockdown of Pol ι significantly reduced the expression of MMPs in KYSE-150 cells (Figure 6A). These results indicated that Pol ι induces MMPs expression in ESCC cells. Since AP-1 stimulates MMP gene transcription [25], we further examined the involvement of the AP-1 pathway in Pol ι-induced MMP expression, using a dual-luciferase reporter gene assay. The pAP1-Luc (Promega, USA) and pRL-SV40 (Promega, USA) vectors were co-transfected into KYSE-150 cells with Pol ι knockdown or ECA-109 cells with Pol ι overexpression. The luciferase activity was determined by an luminometer 36h after transfection. As shown in Figure 6B, overexpression of Pol ι enhances AP-1-mediated reporter activity, whereas knockdown of Pol ι leads to a significant inhibition of the reporter activity. In addition, we observed that overexpression of Pol ι enhances and knockdown of Pol ι diminishes the phosphorylation of JNK, an established critical regulator of AP-1 activation [26] in ESCC cells. To determine the potential role of the JNK-AP-1-MMP signal pathway in Pol ι-induced ESCC cell invasion and metastasis, Pol ι-overexpressing ECA-109 cells were exposed to a specific JNK inhibitor, SP600125. Treatment with SP600125 almost completely inhibited Pol ι-induced JNK activation and MMP overexpression (Figure 7A). Furthermore, SP600125 also prevented Pol ι-enhanced ESCC cell migration and invasion, as determined by wound-healing and the transwell chamber assays (Figure 7B and 7C). Taken together, these results revealed that Pol ι enhances the metastatic potential of ESCC cells via activating the JNK-AP-1-MMPs pathway.

## DISCUSSION

The presence of metastatic cancer cells in the regional lymph nodes is the most important prognostic factor for esophageal cancers [28]. It has been shown that the overall 5-year survival rate after surgery is 70-92% for patients without lymph node metastasis, whereas the survival rate decreases to 18-47% in patients with lymph node metastasis [9, 29]. Thus, identification of biomarkers indicative of local invasion and regional metastasis could be advantageous for ESCC prognosis and management. In the present study, we examined the expression of Pol ι and its association with tumor metastasis in 82 samples of ESCC. Our data demonstrated that the expression level of *Pol ι* is positively correlated with ESCC lymph nodes metastasis (Figure 1D), an observation that is in agreement with our previous conclusion [22]. Consistent with this observation, the expression of Pol ι was significantly associated with a poor prognosis in patients with ESCC (Figure 2). Using well established ESCC cell model systems, we found that Pol ι indeed stimulates the invasiveness and migration of ESCC cells *in vitro*. A tail vein injection of ESCC cells *in vivo* confirmed that Pol ι could enhance the potential for colonization of ESCC cells (Figures 4 and 5). Therefore, the results we obtained from human ESCC tissue samples, ESCC cell lines, and a xenograft mouse model ESCC support the conclusion that Pol ι promotes tumor invasion and metastasis in ESCC, and may serve as a prognostic marker for this devastating malignancy.

MMPs are a family of structurally related zinc-and calcium-dependent endopeptidases that enhance the progression of the epithelial-to-mesenchymal transition (EMT) [30, 31] via degrading various components of extracellular matrix (ECM) and promoting detachment of epithelial cells from the surrounding tissue [32]. MMP-2 and MMP-9 participate in proteolysis of major components of the basement membrane and are associated with tumor invasion and lymph node metastasis [33, 34]. We have demonstrated that the expression levels of *Pol ι* are positively correlated with that of *MMP-2* and *MMP-9* in ESCC tissues, and that Pol ι increases the expression of MMP-2 and MMP-9 in ESCC cell lines (Figure 1E, 1F and Figure 6). These observations provide a potential explanation for the process of Pol ι-induced tumor invasion and metastasis. This insight was further corroborated by the finding that Pol ι significantly enhances transcription of the luciferase reporter gene containing the AP-1 binding element (Figure 6B), suggesting that AP-1 activity is required in Pol ι-induced MMP expression. Note that AP-1 is known to positively regulate the transcription of MMP genes in cancer cells [25].

JNKs are members of the mitogen activated protein kinase (MAPK) family, and are considered to be the crucial upstream kinases involved in AP-1 activation [26]. We found that Pol ι enhances the phosphorylation of JNKs, and that inhibition of JNKs eliminates Pol ι-induced expression of MMP-2/9 (Figure 7). Furthermore, when the JNK inhibitor was applied to the ESCC cells overexpressing Pol ι, the Pol ι-induced aggressive phenotype of enhanced cell motility and invasion was reversed. JNKs were initially identified as stress activated protein kinases (SPAK) and are highly activated by cellular stresses, such as DNA damage [35]. Interestingly, transforming growth factor (TGF) β-activated kinase 1 (TAK1) is activated by the DNA damage response, which in turn, phosphorylates and activates JNK [35, 36]. It remains unclear whether overexpression of Pol ι induces the DNA damage response, subsequently leading to the activation of TAK1-JNK signal pathway. Nonetheless, a recent study by Fu and colleagues suggests that TAK1 expression correlates with lymph node metastasis and is a negative indicator of patient prognosis in resected T3N1-3M0 ESCCs [37]. Further investigation of the the potential interplay between Pol ι and the TAK1-JNK pathway during ESCC progression is warranted. In addition, Akt and other MAPK pathways are also reported to be coupled to MMP-2 and MMP-9 expression [38] and play essential roles during cancer progression. The potential interaction of Pol ι with these signaling pathways also merits further exploration.

In summary, our study has demonstrated that the expression level of Pol ι is positively associated with lymph node metastasisand poor prognosis in ESCC. Mechanistic studies revealed that Pol ι enhances the motility and invasiveness of ESCC cells via JNK-AP-1-mediated upregualtion of MMP2/9. These findings illustrate a role for Pol ι in ESCC progression, indicating that Pol ι is a potential prognostic marker and novel therapeutic target for metastatic ESCC.

## MATERIALS AND METHODS

### Tissue samples and cell lines

Human ESCC tissues and adjacent tissues used in this study were obtained from Nanjing Medical University Affiliated Suzhou Hospital (Jiangsu, China). The tissue samples were immediately snap-frozen and stored at −80°C for real-time PCR analysis and histological examination. All of the samples were obtained with informed consent and the study was approved by the Institutional Ethics Committee of Nanjing Medical University. Human ESCC cell lines, including ECA-109, TE-1, TE-10 and KYSE-150 were obtained from the Shanghai Cell Bank (Shanghai, China). ECA-109, TE-1 and TE-10 cells were cultured in DMEM medium, and KYSE-150 cells were cultured in RPMI-1640 medium. All of the media (Hyclone, USA) were supplemented with 10% FBS (Hyclone, USA). The cells were incubated in a humidified atmosphere, with 5% CO^2^ at 37°C.

### RNA extraction and quantitative RT-PCR

Total RNA was isolated using TRIzol reagent (Invitrogen Life Technologies, Carlsbad, CA, USA) following the manufacturer's instructions. The concentrations of RNA were determined using a NanoDrop2000 (NanoDrop, USA). For reverse transcription, 500 ng of RNA per sample was reverse transcribed using an oligo(dT)_12_ primer and Superscript II reverse transcriptase (Invitrogen Life Technologies, USA). qPCR analyses were conducted to quantitate mRNA expression using SYBR Premix Ex Taq (TaKaRa, Japan) with *β-actin* mRNA level as an internal control. The primers for *β-actin*, *Pol ι*, *MMP-2* and *MMP-9* were as follows: *β-actin*, Forward:5′-AGCGAGCATCCCCCAAAGTT-3′, Reverse: 5′-GGG CACGAAGGCTCATCATT-3′; *Pol ι*, Forward: 5′-ACAA ACCGGGATTTCCTACC-3′, Reverse: 5′-TCACACTTC CTTTCCCTTGAA-3′; *MMP-2*, Forward:5′-ACGGAA AGATGTGGTGTG-3′, Reverse:5′-TGGTGTAGGTGTA AATGGGT-3′; *MMP-9*, Forward: 5′-TATGGTCCTCG CCCTGAACCT-3′, Reverse: 5′-GCACAGTAGTGGC CGTAGAAGG-3′. The results of qPCR were defined by the threshold cycle (Ct), and relative expression levels were calculated by using the 2^−^^ΔCt^ method. PCR was performed using a StepOne Plus instrument (Applied Biosystems, USA).

### Immunohistochemical (IHC) analyses

The expression pattern of Pol ι in human tissue samples was analyzed using immunohistochemistry. Tumor tissue sections were deparaffinized and heattreated with citrate buffer, pH 6.0, for 5 min as an epitope retrieval protocol. The tissue sections were then exposed to 0.03% hydrogen peroxide for 5 min to block endogenous peroxidase activity followed by incubation with POLI antibody (Proteintech, USA) diluted at 1:100 for 2 h at room temperature. HRP-conjugated anti-mouse antibody was then added for 1 h and the color was developed using 3-3′-diaminobenzidine. Following washing, the sections were counterstained with hematoxylin, washed and dipped briefly in a water bath containing drops of ammonia, prior to dehydration and mounting in Diatex. The stained sections were analyzed and scored using a Leica microscope (Leica Corporation, Germany).

### Generation of stable cell lines

The human *Pol ι* coding region was amplified by PCR using a primer pair specific to *Pol ι* and the siRNA targeting *Pol ι* was obtained from Guangzhou RIBOBIO (Guangzhou, China). The fragments were inserted into the lentivirus (LV) expression vectors (containing EGFP, Shanghai GenePharma, China) and packaged into viral particles. The particles were individually used to infect ECA-109 cells and KYSE-150 cells. The cells were then harvested at 3 days post-infection and selected in medium containing 1μg/ml Puromycin (Sigma-Aldrich, USA) for 7 days. The stable cell lines were validated using qRT-PCR and western blot.

### Wound-healing and invasion assays

Wound-healing assay was used to detect cell migration *in vitro*. Cells were seeded in 6-well plates and reached confluence in 24h. A thin mark was drawn vertically with a pipette tip in the 6-well plate. Cells were then washed three times with PBS to remove the floating and detached cells. Fresh serum-free medium was added, and photos were taken at 0, 24, 48 and 96h to assess cell migration using a light microscope (Leica Corporation, Germany).

Cell invasion was assessed using Matrigel-coated Transwell chambers (Costar Inc., UK). Cells were initially cultured in serum-free medium for 12 h. A total of 1×10^5^ (200 μl) cells were then plated in serum-free medium with BSA on 24-well Transwell inserts pre-coated with 40 μl Matrigel (1:4 dilution; BD Bioscience, CA). The lower chambers were filled with medium that contained 10% fetal bovine serum. After incubation for 24h (KYSE-150 cells) or 48 h (ECA-109 cells), the cells remaining in the upper chambers were scraped off, and the invading cells were fixed with 3.7% paraformaldehyde and stained with crystal violet. The penetration of cells through the membrane was photographed under a microscope.

### Western blot analysis

Cells were harvested and lysed in RIPA lysis buffer (Beyotime Biotechnology, China) containing protease inhibitors for 20 min at 4°C. The proteins were separated by 10% SDS-PAGE and transferred to PVDF membranes (Millipore, MA). After blocking with 5% nonfat milk, the membranes were incubated with primary antibodies targeting β-actin (Beyotime Biotechnology, China), Pol ι (Proteintech, USA), MMP-2, MMP-9, JNK (Abcam, USA) and p-JNK (Cell Signaling Technology, USA). The membranes were then incubated with a horseradish peroxidase (HRP)-conjugated anti-rabbit or anti-mouse secondary antibody (Beyotime Biotechnology, China). The protein bands were visualized using enhanced chemiluminescence (ECL, Beyotime Biotechnology, China). Endogenous β-actin protein expression was detected as the internal control for each sample.

### Luciferase reporter gene assay

Pol ι overexpression and knockdown cells grown in 24-cell plates were transfected with 1 μg of pAP1-Luc (Clontech, USA) vector for 36h. Cell lysates were prepared using the lysis buffer contained in the Dual-Luciferase reporter assay kit (Promega, USA) and luciferase activity was measured by a luminometer (Promega-GloMax, USA). Relative luciferase activity was normalized by co-transfection with 50 ng pRL-SV40 vector and analysis of Ranilla luciferase activity.

### *In vivo* imaging of KYSE-150 cell colonization

Four-week old female BALB/c nude mice were purchased from Shanghai SLAC Laboratory Animal Co., Ltd. (Shanghai, China), and maintained under specific pathogen-free conditions. KYSE-150 cells stably expressing shPol ι-EGFP or the vector control were washed from sub-confluent cell culture plates with PBS and then were re-suspended with RPMI-1640 at a concentration of 2×10^6^ cells/ml. A 0.15 ml of the suspended KYSE-150 cells was intravenously injected into the tail vein of each nude mouse. After 24 h, 48 h and 30 days, KYSE-150 cell colonization in the nude mouse organs was visualized using the Kodak *in vivo* imaging system. All the photos were taken under the same condition. The relative fluorescence intensity in the tissues was calculated using the Image J image analysis software (MD, USA). At 30 days, the livers, kidneys and lungs of nude mice were removed and fixated in 10% phosphate-buffered formaldehyde, and embedded in paraffin. Tumors were confirmed in H&E-stained sections. The design and implementation of the study were approved by the Ethics Committee at Nanjing Medical University.

### Statistical analysis

The quantitative results are presented as the mean values ± SEM. Statistical analyses were performed using SPSS 19.0 software (IBM, USA). Statistical significance was considered to be a P-value < 0.05. Differences between groups were estimated using the χ2 test and Student's t test. Overall survival rate was calculated actuarially according to the Kaplan-Meier method and analyzed by the log-rank test. Relationships of variables were explored by Pearson's correlation.

## References

[1] Lagergren J, Lagergren P (2013). Recent developments in esophageal adenocarcinoma. CA Cancer J Clin.

[2] Song Y, Li L, Ou Y, Gao Z, Li E, Li X, Zhang W, Wang J, Xu L, Zhou Y, Ma X, Liu L, Zhao Z, Huang X, Fan J, Dong L (2014). Identification of genomic alterations in oesophageal squamous cell cancer. Nature.

[3] Conteduca V, Sansonno D, Ingravallo G, Marangi S, Russi S, Lauletta G, Dammacco F (2012). Barrett's esophagus and esophageal cancer: an overview. International journal of oncology.

[4] Schweigert M, Dubecz A, Stein HJ (2013). Oesophageal cancer--an overview. Nature reviews Gastroenterology & hepatology.

[5] Giancotti FG (2013). Mechanisms governing metastatic dormancy and reactivation. Cell.

[6] Christofori G (2006). New signals from the invasive front. Nature.

[7] Xia Y, Lian S, Khoi PN, Yoon HJ, Joo YE, Chay KO, Kim KK, Do Jung Y (2015). Chrysin inhibits tumor promoter-induced MMP-9 expression by blocking AP-1 via suppression of ERK and JNK pathways in gastric cancer cells. PloS one.

[8] Cho JW, Choi SC, Jang JY, Shin SK, Choi KD, Lee JH, Kim SG, Sung JK, Jeon SW, Choi IJ, Kim GH, Jee SR, Lee WS, Jung HY (2014). Lymph Node Metastases in Esophageal Carcinoma: An Endoscopist's View. Clinical endoscopy.

[9] Wu SG, Sun JY, Yang LC, Zhou J, Li FY, Li Q, Lin HX, Lin Q, He ZY (2015). Prognosis of patients with esophageal squamous cell carcinoma after esophagectomy using the log odds of positive lymph nodes. Oncotarget.

[10] Abbas T, Keaton MA, Dutta A (2013). Genomic instability in cancer. Cold Spring Harbor perspectives in biology.

[11] Hoeijmakers JH (2001). Genome maintenance mechanisms for preventing cancer. Nature.

[12] Ghosal G, Chen J (2013). DNA damage tolerance: a double-edged sword guarding the genome. Translational cancer research.

[13] Sharma S, Helchowski CM, Canman CE (2013). The roles of DNA polymerase zeta and the Y family DNA polymerases in promoting or preventing genome instability. Mutation research.

[14] Makridakis NM, Reichardt JK (2012). Translesion DNA polymerases and cancer. Frontiers in genetics.

[15] Stallons LJ, McGregor WG (2010). Translesion synthesis polymerases in the prevention and promotion of carcinogenesis. Journal of nucleic acids.

[16] Lee GH, Matsushita H (2005). Genetic linkage between Pol iota deficiency and increased susceptibility to lung tumors in mice. Cancer science.

[17] Iguchi M, Osanai M, Hayashi Y, Koentgen F, Lee GH (2014). The error-prone DNA polymerase iota provides quantitative resistance to lung tumorigenesis and mutagenesis in mice. Oncogene.

[18] Ohkumo T, Kondo Y, Yokoi M, Tsukamoto T, Yamada A, Sugimoto T, Kanao R, Higashi Y, Kondoh H, Tatematsu M, Masutani C, Hanaoka F (2006). UV-B radiation induces epithelial tumors in mice lacking DNA polymerase eta and mesenchymal tumors in mice deficient for DNA polymerase iota. Molecular and cellular biology.

[19] Pan Q, Fang Y, Xu Y, Zhang K, Hu X (2005). Down-regulation of DNA polymerases kappa, eta, iota, and zeta in human lung, stomach, and colorectal cancers. Cancer letters.

[20] Yang J, Chen Z, Liu Y, Hickey RJ, Malkas LH (2004). Altered DNA polymerase iota expression in breast cancer cells leads to a reduction in DNA replication fidelity and a higher rate of mutagenesis. Cancer research.

[21] Zhou J, Zhang S, Xie L, Liu P, Xie F, Wu J, Cao J, Ding WQ (2012). Overexpression of DNA polymerase iota (Poliota) in esophageal squamous cell carcinoma. Cancer science.

[22] Sun H, Zou S, Zhang S, Liu B, Meng X, Li X, Yu J, Wu J, Zhou J (2015). Elevated DNA polymerase iota (Poli) is involved in the acquisition of aggressive phenotypes of human esophageal squamous cell cancer. International journal of clinical and experimental pathology.

[23] Kayani B, Zacharakis E, Ahmed K, Hanna GB (2011). Lymph node metastases and prognosis in oesophageal carcinoma--a systematic review. European journal of surgical oncology.

[24] Fingleton B (2006). Matrix metalloproteinases: roles in cancer and metastasis. Frontiers in bioscience.

[25] Chakraborti S, Mandal M, Das S, Mandal A, Chakraborti T (2003). Regulation of matrix metalloproteinases: an overview. Molecular and cellular biochemistry.

[26] Sehgal V, Ram PT (2013). Network Motifs in JNK Signaling. Genes Cancer.

[27] Valastyan S, Weinberg RA (2011). Tumor metastasis: molecular insights and evolving paradigms. Cell.

[28] Karaman S, Detmar M (2014). Mechanisms of lymphatic metastasis. The Journal of clinical investigation.

[29] Jin H, Qiao F, Chen L, Lu C, Xu L, Gao X (2014). Serum metabolomic signatures of lymph node metastasis of esophageal squamous cell carcinoma. Journal of proteome research.

[30] Ebelt ND, Cantrell MA, Van Den Berg CL (2013). c-Jun N-Terminal Kinases Mediate a Wide Range of Targets in the Metastatic Cascade. Genes Cancer.

[31] van Zijl F, Krupitza G, Mikulits W (2011). Initial steps of metastasis: cell invasion and endothelial transmigration. Mutation research.

[32] Radisky ES, Radisky DC (2010). Matrix metalloproteinase-induced epithelial-mesenchymal transition in breast cancer. Journal of mammary gland biology and neoplasia.

[33] Li Y, Ma J, Guo Q, Duan F, Tang F, Zheng P, Zhao Z, Lu G (2009). Overexpression of MMP-2 and MMP-9 in esophageal squamous cell carcinoma. Diseases of the esophagus.

[34] Zhao G, Kang J, Jiao K, Xu G, Yang L, Tang S, Zhang H, Wang Y, Nie Y, Wu K, Fan D, Zhang D (2015). High Expression of GRP78 Promotes Invasion and Metastases in Patients with Esophageal Squamous Cell Carcinoma. Digestive diseases and sciences.

[35] Picco V, Pages G (2013). Linking JNK Activity to the DNA Damage Response. Genes Cancer.

[36] Yu Y, Ge N, Xie M, Sun W, Burlingame S, Pass AK, Nuchtern JG, Zhang D, Fu S, Schneider MD, Fan J, Yang J (2008). Phosphorylation of Thr-178 and Thr-184 in the TAK1 T-loop is required for interleukin (IL)-1-mediated optimal NFkappaB and AP-1 activation as well as IL-6 gene expression. The Journal of biological chemistry.

[37] Wen J, Hu Y, Luo KJ, Yang H, Zhang SS, Fu JH (2013). Positive transforming growth factor-beta activated kinase-1 expression has an unfavorable impact on survival in T3N1-3M0 esophageal squamous cell carcinomas. The Annals of thoracic surgery.

[38] Lee KR, Lee JS, Song JE, Ha SJ, Hong EK (2014). Inonotus obliquus-derived polysaccharide inhibits the migration and invasion of human non-small cell lung carcinoma cells via suppression of MMP-2 and MMP-9. International journal of oncology.

